# Preoperative Nutritional Status Contributes to the Development of Neutropenia Event in Patients With Gastric Cancer Receiving CAPEOX Adjuvant Chemotherapy

**DOI:** 10.3389/fonc.2020.00692

**Published:** 2020-04-30

**Authors:** Ai Tang Xiao, Yi Xin Tong, Xiang Shang Xu, Yi Zhou, Sheng Zhang

**Affiliations:** Department of Gastrointestinal Surgery, Tongji Medical College, Tongji Hospital, Huazhong University of Science and Technology, Wuhan, China

**Keywords:** CAPEOX chemotherapy, neutropenia, risk factor, prealbumin, PNI

## Abstract

**Purpose:** The aim of this study is to evaluate the risk factors for ≥ grade 3 neutropenia in gastric cancer patients receiving postoperative adjuvant chemotherapy.

**Methods:** This is a retrospective study from a single tertiary referral hospital. Patients diagnosed with gastric cancer who met the inclusion criteria were included in this study. Baseline and clinicopathological characteristics of the patients were collected. Patients were followed-up for 12 months and the incidence of neutropenia were recorded. Factors associated with neutropenia of chemotherapy in cycle 1 were investigated.

**Results:** A total of 202 patients with gastric cancer were included. All patients received oxaliplatin plus oral capecitabine (CAPEOX) as the adjuvant chemotherapy. The incidence of ≥ grade 3 neutropenia is 11.9% (24/202) in cycle 1 among all patients. In multivariate analysis, independent risk factors for ≥ grade 3 neutropenia were serum prealbumin level (*p* = 0.041), prognostic nutritional index (PNI) (*p* = 0.049) and pre-cycle neutrophil count (*p* = 0.007).

**Conclusions:** Our findings for the first time showed that nutritional parameter as prealbumin level and PNI are independent risk factors for neutropenia in gastric cancer patients receiving adjuvant chemotherapy. This may provide evidence for further investigation on prophylaxis use of granulocyte colony-stimulating factor in selected high-risk patients to prevent sever neutropenia in cycle 1 of adjuvant chemotherapy.

## Introduction

Gastric cancer (GC) is a common cancer type of gastrointestinal system and leading cause of cancer death in the world (1). Surgery together with adjuvant chemotherapy (AC) remains the standard treatment for stage III and selected stage II patients (2–4). Recently updated National Comprehensive Cancer Network (NCCN) guidelines recommended that CAPEOX/XELOX (oxaliplatin plus oral capecitabine) as the first-line adjuvant chemotherapy regimens for patients with curatively resected gastric cancer (5). In China, CAPEOX/XELOX was also recommended as the first-line adjuvant chemotherapy agents for gastric cancer by the 2019 Guidelines of Chinese Society of Clinical Oncology (CSCO) (6).

Chemotherapy may induce various toxicities such as vomit, diarrhea, neurotoxicity and neutropenia. Chemotherapy-induced neutropenia (CIN) is one of the most important dose-limiting toxicities which is associated with an increased risk of infection and mortality (7). Multiple factors including patient demographics such as old age and female gender, decreased performance status, poor nutrition status, low baseline absolute neutrophil count (ANC) may correlate with increased risk of CIN (8, 9). Interventions to prevent CIN include dose reduction of chemotherapy reagents and prophylactic use of colony-stimulating factors (CSFs). Current guidelines recommend prophylactic use of CSFs in patients with a >20% risk of febrile neutropenia (FN) (10, 11). However, it is still challenging for clinical decision on selecting proper patients for prophylactic use of CSFs to prevent CIN.

Adjuvant CAPEOX/XELOX is routinely given as six to eight courses (3 weeks/course) for 6 months. It has been reported that the incidence of ≥ grade 3 neutropenia is relatively rare during CAPEOX/XELOX treatment, which is around 8.6–12.6% (12, 13). Although some studies reported predictive model of CIN induced by chemotherapy in various cancer type, there is currently no study exclusively identifying risk factors for neutropenia in gastric cancer patients after CAPEOX/XELOX treatment (14–16). Thus, this study aims to identify potential risk factors for prediction of ≥ grade 3 neutropenia in cycle 1 of adjuvant chemotherapy in gastric cancer patients.

## Methods

### Study Design and Patients

In a single tertiary referral hospital, data from patients with gastric cancer who underwent adjuvant chemotherapy between January 2017 and May 2019 were retrospectively collected and analyzed. Patients aged over 18 years who met the following inclusion criteria were recruited in the study: (1) histopathological confirmed diagnosis of gastric cancer; (2) patients underwent operation as gastrectomy with D2 lymph node dissection. (3) patients with an Eastern Cooperative Oncology Group (ECOG) performance status (PS) of 0 or 1; (17) (4) age<75 years old; (5) had no prior history of chemotherapy or radiotherapy; (6) have no evidence of bone marrow metastasis; (7) have a relative dose intensity (RDI) of oxaliplatin and capecitabine >90% and without any dose reduction during the first circle.

The exclusion criteria were (1) synchronously diagnosis of other malignancies; (2) patients already received neo-adjuvant therapy; (3) had incomplete records of data.

Ethical approval for the study was obtained from the institutional medical ethics committee, with all aspects in this study complying with the Helsinki Declaration. Informed consent of chemotherapy was signed by all participants.

### Data Collection

Relevant data were retrospectively collected from the electronic medical record system. The following information was collected for analysis: 1. Demographic characteristics such as age, gender, body mass index (BMI), education status, socioeconomic status, smoking or alcohol; 2. Laboratory characteristics such as albumin, prealbumin, total cholesterol, absolute neutrophil count (ANC), absolute lymphocyte count (ALC), platelet count, hemoglobin, estimated glomerular filtration rate (eGFR), serum tumor markers as carcinoembryonic antigen (CEA) and carbohydrate antigen 19-9 (CA19-9), 3. Clinical characteristics such as tumor size, differentiation, invasion depth (T), presence of lymph node metastases (*N*); 4. Perioperative characteristics such as surgical approach, postoperative complication (according to Clavien-Dindo criteria) (18), length of stay, initiation timing of chemotherapy.

### Study Endpoint and Assessment of Neutropenia

All included patients received at least one cycle of CAPEOX/XELOX chemotherapy, with an intravenous infusion of oxaliplatin (130 mg/m^2^) on day 1 and oral capecitabine of (1,000 mg/m^2^ twice daily) on days 2–14, 21 days as one cycle. The primary endpoint of this study was the incidence of ≥ grade 3 neutropenia in cycle 1 of the adjuvant chemotherapy. No prophylactic G-CSF was applied before cycle 1.

Patients were followed up after discharged once a week and made additional visits based on adverse effects. Blood samples were collected at the end of 21-day cycle. Grades of neutropenia were determined based on Common Terminology Criteria for Adverse Events (CTCAE, version 3.0) of National Cancer Institute (NCI), with grades 1–4 neutropenia defined as 1.5–2.0^*^10^9^/L, 1.0–1.5^*^10^9^/L, 0.5–1.0^*^10^9^/L and <0.5^*^10^9^/L, respectively (19).

### Statistical Analyses

In general, all continuous variables were presented as mean (standard deviation, SD)/medians (range) and analyzed with Mann-Whitney U test. Categorical variables were reported as whole numbers and percentages and analyzed with chi-square test or Fisher's exact test. The lower limit of the normal reference range (200 mg/L) in our hospital was used to determine the cut-off value of serum prealbumin level as “reduced” and “normal.” The prognostic nutritional index (PNI) was calculated as follows: 10×serum albumin (g/dl) +0.005×lymphocyte count (per mm^3^). The receiver operation curve (ROC curve) method was used to determine the cut-off value of PNI as “low” and “normal.” The univariate logistic regression was used to evaluate potential risk factors for ≥ grade 3 neutropenia. Only factors with *p*-value < 0.1 in univariate analysis were included in the final multivariate analysis model. Multivariate logistic regression was employed to identify independent risk factors for ≥ grade 3 neutropenia. All *p*-values were reported as two-sided with a significance level of 0.05. All statistical tests were performed in SPSS version 21.0 (IBM, Armonk, NY, USA).

## Results

### Baseline and Clinic-Pathological Characteristics

Between January 2017 and May 2019, 256 gastric cancer patients underwent operation were reviewed and considered eligible for this study. After screening based on inclusion and exclusion criteria, 202 patients received at least 1 cycle of adjuvant chemotherapy were included in final study. The mean age of included patients was 52.6 ± 10.8 comprising 126 (62.4%) men and 76 (37.6%) women. 60.9 and 39.1% of patients were with stage II or III disease, respectively. 96.5% patients underwent laparoscopic gastrectomy and 3.5% patients underwent open gastrectomy. 13.4% (27/202) patients suffered from Grade 1&2 post-operative complications. The median time between adjuvant chemotherapy and operation was 29 ± 10 days. Other information was summarized in Table 1.

**Table 1:** The demographic and clinical characteristics of patients in cycle 1.

**Variables**	**All patients (*n* = 202)**	**With < Grade 3 neutropenia (*n* = 178)**	**With ≥Grade 3 neutropenia (*n* = 24)**	***p*-value**
**PATIENT FACTORS**				
Age, mean (SD), y	52.6 ± 10.8	52.3 ± 11.1	54.9 ± 7.9	0.255
Gender, Male, *n* (%)	126 (62.4%)	111 (62.4%)	15 (62.5%)	0.989
Body mass index mean (SD), kg/m^2^	22.5 ± 3.8	22.8 ± 2.7	22.3 ± 4.1	0.895
**Education**				
Illiterate/primary or high school	89.6% (181/202)	89.9% (160/178)	87.5% (21/24)	0.719
Undergraduate or higher	10.4% (21/202)	10.1% (18/178)	12.5% (3/24)	
**Socioeconomic status**				
Low to moderate income	86.1% (174/202)	84.8% (151/178)	95.8% (20/24)	0.143
High income	13.9% (28/202)	15.2% (27/178)	4.2% (1/24)	
**Smoking**				
Yes	23.8% (48/202)	24.7% (44/178)	16.7% (4/24)	0.384
No	76.2% (154/202)	75.3% (134/178)	83.3% (20/24)	
**Alcohol**				
Yes	12.4% (25/202)	11.8% (21/178)	16.7% (4/24)	0.497
No	87.6% (177/202)	88.2% (157/178)	83.3% (20/24)	
**LABORATORY FACTORS**				
Albumin (g/L), mean (SD)	41.0 ± 3.85	41.4 ± 3.62	38.1 ± 4.15	**0.001**
Prealbumin (mg/L), mean (SD)	221.1 ± 51.0	224.7 ± 52.9	194.3 ± 17.1	**0.006**
Absolute neutrophil count (*10^9^/L), mean (SD)	3.51 ± 1.57	3.56 ± 1.60	3.15 ± 1.36	0.231
Absolute lymphocyte count (*10^9^/L), mean (SD)	1.66 ± 0.6	1.70 ± 0.7	1.32 ± 0.4	**0.007**
Platelet count (*10^9^/L), mean (SD)	255.0 ± 89.0	258.8 ± 91.3	227.1 ± 63.5	0.101
Hemoglobin (g/L), mean (SD)	120.6 ± 25.5	122.2 ± 23.7	108.8 ± 34.4	**0.015**
eGFR (mL/min), mean (SD)	94.3 ± 16.4	94.3 ± 16.7	94.4 ± 14.4	0.990
**Serum CEA**				
Normal	69.3% (140/202)	69.7% (124/178)	66.7% (16/24)	0.765
Elevated	30.7% (62/202)	30.7% (54/178)	33.3% (8/24)	
**Serum prealbumin**				
≥200 mg/L	64.9% (131/202)	69.1% (123/178)	33.3% (8/24)	**0.001**
<200 mg/L	35.1% (71/202)	30.9% (55/178)	66.7% (16/24)	
**PNI**				
≥51.2	39.1% (79/202)	43.3% (77/178)	8.3% (2/24)	**0.001**
<51.2	60.9% (123/202)	56.7% (101/178)	91.7% (22/24)	
**DISEASE FACTORS**				
**TNM stage**				
II	60.9% (123/202)	61.8% (110/178)	54.2% (13/24)	0.472
III	39.1% (79/202)	38.2% (68/178)	45.8% (11/24)	
**Lymph node metastasis**				
No	38.6% (78/202)	41.0% (73/178)	16.7% (4/24)	**0.021**
Yes	61.4% (124/202)	59.0% (105/178)	83.3% (20/24)	
Tumor size (cm), mean (SD)	4.1 ± 1.9	3.8 ± 1.7	6.0 ± 2.6	**0.001**
**Differentiation**				
High/moderate	47.5% (96/202)	51.1% (91/178)	20.8% (5/24)	**0.005**
Poor	52.5% (106/202)	48.9% (87/178)	79.2% (19/24)	
**PERIOPERATIVE FACTORS**				
**Operation**				
Laparoscopic	96.5% (195/202)	96.6% (172/178)	95.8% (23/24)	0.611
Open	3.5% (7/202)	3.4% (6/178)	4.2% (1/24)	
**Complications**				
No	86.6% (175/202)	87.1% (155/178)	83.3% (20/24)	0.613
Grade 1 & 2	13.4% (27/202)	12.9% (23/202)	16.7% (4/24)	
Time between AC and operation, median, days	29 ± 10	30 ± 11	26 ± 2	0.135
**Neutropenia in cycle 1**				
No	48.0% (97/202)	N/A	N/A	N/A
Grade 1	27.2% (55/202)			
Grade 2	12.9% (26/202)			
Grade 3	10.9% (22/202)			
Grade 4	1.0% (2/202)			

*For p-value: Boldface type indicates significant difference*.

*SD, standard deviation; eGFR, estimated glomerular filtration rate; CEA, carcinoembryonic antigen; TNM, tumor-node-metastasis; AC, adjuvant chemotherapy; PNI, prognostic nutritional index; N/A, not available*.

### Prevalence of Neutropenia in Cycle 1

In cycle 1, the sum incidence of neutropenia of all grades was 52% (105/202). The incidence of ≥ grade 3 neutropenia was 11.9% (24/202). The demographic characteristics of patients with or without ≥ grade 3 neutropenia are shown in Table 1. Compared to patients with no neutropenia or grade 1–2 neutropenia in cycle 1, most patients with ≥ grade 3 neutropenia have decreased prealbumin level and PNI score (Table 1).

### Risk Factors Associated With ≥Grade 3 Neutropenia in Cycle 1

Risk factors for development of ≥ grade 3 neutropenia in cycle 1 of adjuvant chemotherapy identified from univariate analysis were shown in Table 2. The results showed that socioeconomic status (low or moderate income vs. high income, HR = 4.11, *p* = 0.18), serum albumin level (<35 vs. ≥35 g/L, HR = 9.11, *p* = 0.001), serum prealbumin level (<200 vs. ≥200 mg/L, HR = 4.47, *p* = 0.001), preoperative neutrophil count (<2.0^*^10^9^/L vs. ≥2.0^*^10^9^/L, HR = 4.48, *p* = 0.004), PNI (<51.2 vs. ≥51.2, HR=8.39, *p* = 0.005), differentiation (poor vs. high/moderate, HR = 3.98, *p* = 0.009) and lymph node metastasis (yes vs. no, HR = 3.48, *p* = 0.028) were significantly associated with ≥ grade 3 neutropenia in cycle 1.

**Table 2:** Univariate and multivariate analysis for factors effecting.

**Variable**	**Univariate HR (95%CI)**	***p*-value**	**Multivariate HR (95%CI)**	***p*-value**
**Age**				
<65	1 (Ref)	0.45		
≥65	1.67 (0.44–6.31)			
**Gender**				
Male	1 (Ref)	0.99		
Female	1.00 (0.44–6.31)			
**Body mass index**				
≥20	1 (Ref)	0.78		
<20	0.95 (0.68–3.21)			
**Socioeconomic status**				
High income	1 (Ref)	**0.18**	1 (Ref)	0.22
Low to moderate income	4.11 (0.53–31.74)		3.73 (0.45–31.25)	
**Education**				
High school/undergraduate	1 (Ref)	0.72		
Illiterate/primary school	1.27 (0.35–4.68)			
**Smoking**				
No	1 (Ref)	0.39		
Yes	0.61 (0.20–1.88)			
**Alcohol**				
No	1 (Ref)	0.50		
Yes	1.51 (0.47–1.80)			
**Albumin**				
≥35 g/L	1 (Ref)	**0.001**	1 (Ref)	0.055
<35 g/L	9.11 (2.42–34.33)		4.161 (0.97–17.87)	
**Prealbumin**				
≥200 mg/L	1 (Ref)	**0.001**	1 (Ref)	**0.041**
<200 mg/L	4.47 (1.81–11.07)		2.89 (1.05–7.99)	
**Preoperative neutrophil count**				
≥2.0*10^9^/L	1 (Ref)	**0.004**	1 (Ref)	**0.007**
<2.0*10^9^/L	4.48 (1.61–12.49)		5.00 (1.54–16.25)	
**Preoperative lymphocyte**				
≥1.1*10^9^/L	1 (Ref)	0.681		
<1.1*10^9^/L	0.77 (0.21–2.74)			
**Preoperative hemoglobin**				
≥110 g/L	1 (Ref)	0.334		
<110 g/L	1.57 (0.63–12.49)			
**Serum CEA**				
Normal	1 (Ref)	0.765		
Elevated	1.15 (0.46–2.84)			
**Renal function**				
Normal	1 (Ref)	0.367		
Dysfunction	0.62 (0.22–1.75)			
**PNI**				
≥51.2	1 (Ref)	**0.005**	1 (Ref)	**0.049**
<51.2	8.39 (1.91–36.75)		4.76 (1.01–22.49)	
**Differentiation**				
High/moderate	1 (Ref)	**0.009**	1 (Ref)	0.230
Poor	3.98 (1.42–11.11)		2.04 (0.64–6.50)	
**TNM stage**				
II	1 (Ref)	0.473		
III	1.40 (0.58–3.23)			
**Lymph node metastasis**				
No	1 (Ref)	**0.028**	1 (Ref)	0.228
Yes	3.48 (1.14–10.59)		2.14 (0.62–7.42)	
**Complication**				
No	1 (Ref)	0.614		
Yes	1.348 (0.42–4.30)			

*For p-value: Boldface type indicates significant difference*.

*HR, hazard ratio;SD, standard deviation; eGFR, estimated glomerular filtration rate; CEA, carcinoembryonic antigen; TNM, tumor-node-metastasis; AC, adjuvant chemotherapy; PNI, prognostic nutritional index*.

In the multivariate survival analysis, prealbumin, preoperative neutrophil count and PNI were identified as independent risk factors associated with ≥ grade 3 neutropenia in cycle 1 of adjuvant chemotherapy, with the hazard ratio (HR) of 2.89 (*p* = 0.041, 95%CI 1.05–7.99), 5.00 (*p* = 0.007, 95%CI 1.54–16.25), and 4.76(*p* = 0.049, 95%CI 1.01–22.49) respectively. The details of multivariate analysis were listed in Table 2.

## Conclusions

### Discussion of the Results

In this study, we performed a large retrospective study to investigate the incidence of neutropenia among gastric cancer patients receiving adjuvant chemotherapy in cycle 1. Meanwhile, we also evaluated demographic and clinical characteristics related to development of neutropenia of grade 3 or higher, which may provide evidence for management of chemotherapy and prophylactic application of CSFs in high risk patients. Our results demonstrated that around 11.9% (24/202) patients developed ≥ grade 3 neutropenia in cycle 1 of adjuvant chemotherapy. Furthermore, we for the first time showed that preoperative neutrophil count and nutritional parameters as serum prealbumin level and PNI are independent risk factors for ≥ grade 3 neutropenia in cycle 1 of adjuvant chemotherapy.

### Neutropenia Is a Common Complication of Chemotherapy

Gastric cancer is one of the leading causes of cancer-related death and is an important health problem worldwide. Gastric cancers are highly heterogeneous with respect to histology, differentiation and molecular carcinogenesis. Adequate surgical (R0) resection is the only curative treatment for gastric cancer. Adjuvant and neoadjuvant chemotherapy therapies are standard treatments to improve disease-free survival and overall survival in patients after curative surgery (20). Fluoropyrimidine and platinum regimen are first-line adjuvant chemotherapy for advanced gastric cancer. Capecitabine (Xeloda, Roche) plus oxaliplatin (CAPEOX/XELOX), most common first-line chemotherapy regimens for gastric cancer, were reported to be effective with less adverse effect (21).

Our study showed that the incidence of neutropenia of all grade in cycle 1 is 52% (105/202) and the incidence of grade 3–4 neutropenia 11.9% (24/202) for gastric cancer patients receiving CAPEOX/XELOX adjuvant chemotherapy (Table 1). According to various reports, the incidence of grade 3–4 neutropenia throughout all cycles in patients receiving CAPEOX or FOLFOX adjuvant chemotherapy is around 15–22% (21, 22). Specially targeted on the incidence of ≥grade 3 neutropenia in cycle 1 of CAPEOX/XELOX regimen in gastric cancer patients addressed the importance of our study. Although the incidence is relatively low, it is still certain proportion of patients suffered from ≥ grade 3 neutropenia and the relevant complications (23–25).

### Malnutrition and Decreased Preoperative Neutrophil Count Are Risk Factors for Neutropenia

The most crucial factors for developing neutropenia are the chemotherapy regimens and drug dosage. Besides different treatment regimens, several factors such as older age, advanced stage, impaired kidney function, impaired patients' performance status, decreased pretreatment absolute neutrophil counts and serum albumin level were reported to be associated with severe neutropenia or FN (7, 8). Seo et al. (26) reported that postoperative malnutrition was common in patients after gastrectomy. Postoperative hypoalbuminemia was an independent risk factor for grade 3/4 hematological adverse events during chemotherapy (27). Predictive models were also constructed and validated to discriminated patients with high risk to develop FN (14, 27). However, none of these specially targeted on gastric cancer patients receiving adjuvant chemotherapy. To the best of our knowledge, the present study is the first to identify risk factors to predicts the occurrence of neutropenia (grade 3 or higher) in patients with gastric cancer. In our study we proved that preoperative absolute neutrophil count nadir is an independent predictive factor (HR = 5.00, 95%CI 1.54–16.25) for ≥ grade 3 neutropenia in cycle 1 of adjuvant chemotherapy. Several retrospective studies (28, 29) using a variety of chemotherapies also demonstrated that first-cycle nadir ANC is a primary predictor of neutropenia-related events in cancer patients.

In addition, we showed that patients' nutritional status may have impact on neutropenia event in cycle 1 of adjuvant chemotherapy. It has been widely studied that patients' nutritional status correlated with long-term survival in various cancer. Parameters such as physical data, weight loss, body mass index (BMI), serum albumin and prealbumin level, Glasgow Prognostic Score, Controlling Nutritional Status and PNI are useful tools to evaluate patients' nutritional status (30, 31). Prealbumin, also called transthyretin, is an acute-phase liver protein which has a short half-life as 2–3 days. Prealbumin has been confirmed to reflect liver function and to be significantly associated with inflammation than albumin due to its relatively short half-life. It has also been reported that prealbumin is a prognostic factor for various gastrointestinal malignancies such as colorectal cancer, gastric cancer and hepatocellular carcinoma (32–34). The prognostic nutritional index (PNI), which is based on serum albumin and absolute peripheral lymphocyte count, was proved to be a predictive factor for postoperative complication and poor prognosis factor in gastric cancer patients (35, 36). The correlation between PNI and adverse event in gastric cancer patients receiving chemotherapy has not been reported.

In this retrospective study we identified preoperative serum prealbumin level (HR = 2.89, *p* = 0.041) and PNI (HR = 4.76, *p* = 0.049) as independent risk factors for predicting ≥ grade 3 neutropenia event in cycle 1 of gastric cancer patients receiving adjuvant chemotherapy. Besides, serum albumin level was correlated to ≥grade 3 neutropenia event in univariate analysis while this correlation is not significant in multivariate analysis (*p* = 0.055) (Table 2). The potential reason for this association is that gastric cancer patients that suffered from malnutrition may have impaired immune function. The immune function may play a crucial role in proliferation and differentiation of the neutrophils (37). As a result, impaired immune function may lead to deceased neutrophil count under myelosuppressive chemotherapy.

### Clinical Implication and Limitations

Neutropenia is associated with delay of chemotherapy, increased risk of infection and sometimes can be life-threatening (38). The current guidelines suggest that patients with a high risk (>20%) of FN should receive G-CSF as primary prophylaxis. Patients with an intermediate risk (10–20%) of FN plus at least one risk factor should consider receiving prophylactic G-CSF in each cycle of chemotherapy (10, 11, 39). However, there is no specific study focus on risk factors for neutropenia of CAPEOX/XELOX regimen in gastric cancer patients. This study will provide evidence of selecting patients with risk factor of developing ≥ grade 3 neutropenia event in cycle 1 for prophylactic G-CSF.

The present study has several limitations that should be taken into consideration. First, due to retrospective setting, several data which might contribute to the development of neutropenia are lacking. Second, as a result of small sample size, PNI and prealbumin were borderline for being significant on multivariate analysis. Therefore, a prospective study with a larger sample size and more clinical-pathological measurements are needed to validate the finding of our study and further explore more potential risk factors for development of neutropenia.

## Conclusions

First, our study for the first time indicated that preoperative neutrophil count and nutritional status as prealbumin level and PNI are significantly associated with ≥ grade 3 neutropenia event in cycle 1 of gastric cancer patients receiving CAPEOX/XELOX adjuvant chemotherapy. Second, our finding suggested that proportion of patients will develop severe neutropenia in real world setting therefore it is necessary to identify patients with risk factor for intense observation and prophylactic application of G-CSF.

## Data Availability Statement

The database used and/or analyzed during the current study is not publicly available (to maintain privacy) but can be available from the corresponding author on reasonable request.

## Ethics Statement

This study was approved by the Ethics committee of Tongji Hospital, Tongji Medical College, Huazhong University of Science and Technology. All procedures followed in this study were in accordance with the 1964 Helsinki Declaration and later versions. Informed consent was obtained from patients involved.

## Author Contributions

AX and SZ conceived the study, analyzed the data, and drafted the manuscript. XX and YT helped critically revise the manuscript for important intellectual content. YZ helped collect data. All authors have agreed on the final version and meet the major criteria recommended by the ICMJE http://www.icmje.org/ and participated in the study design.

## Conflict of Interest

The authors declare that the research was conducted in the absence of any commercial or financial relationships that could be construed as a potential conflict of interest.
